# An optimized method for Oil Red O staining with the salicylic acid ethanol solution

**DOI:** 10.1080/21623945.2023.2179334

**Published:** 2023-02-24

**Authors:** Junbao Du, Li Zhao, Quan Kang, Yun He, Yang Bi

**Affiliations:** a Stem Cell Biology and Therapy Laboratory, Children’s Hospital of Chongqing Medical University, Chongqing, P.R. China; b National Clinical Research Center for Child Health and Disorders, Ministry of Education Key Laboratory of Child Development and Disorders, Chongqing Key Laboratory of Pediatrics, Chongqing, P.R. China

**Keywords:** Oil red O staining, ethanol, salicylic acid, isopropanol, lipid droplet

## Abstract

Oil Red O (ORO) staining is a commonly used experimental technique to detect lipid content in cells or tissues. Freshly prepared ORO in 60% isopropanol is the most widely used method at present. However, isopropanol is volatile and harmful to the human body. It will also affect the interpretation of the results due to the formation of crystals and non-specific diffuse staining. In this paper, by screening and validation, we report a salicylic acid ethanol solution (containing 50% ethanol, 5%-10% salicylic acid) for the preparation of ORO solution, which has a better staining effect on lipid staining in cells and tissues, with a clean background and short dyeing time. What’s more, this ORO solution is non-toxic, convenient to prepare, and can be stored for a long time. Therefore, it is reliable, easy to operate, and can be widely popularized and applied in laboratories.

## Introduction

Oil Red O (ORO) is a fat-soluble dye used to stain neutral lipids, cholesteryl esters, and lipoproteins [1,2]. It has been widely used for intracellular lipid staining and tissue staining due to its cellular permeability, neutral fats, mainly triglycerides, can be stained with an orange-red tint [3–6].

The preparation of the dyeing solution is very important to the dyeing effect of ORO. ORO is easily soluble in benzene, soluble in chloroform, acetic acid, ether, ethanol, acetone, petroleum ether, non-volatile oil, hot glycerol, volatile oil, and other organic solvents, but insoluble in water [7,8]. Most organic solvents are volatile and harmful to the human body. At the same time, precipitates of ORO are easily formed after being prepared in such organic solvents, it is easy to leave red crystals on the tissue sample during staining, which affects the interpretation of the results [9]. Among the organic solvents, 100% isopropanol-saturated ORO solution is convenient to store and is the most conventional commercial reagent. When used, ORO dye solution in 60% isopropanol is freshly prepared by diluting in ddH_2_O [2,8,10]. This commonly used method is convenient to operate and has a good staining effect. However, as an organic solvent, isopropanol is volatile, and inhalation of isopropanol is harmful to the human body during operation [11,12]. Therefore, it is necessary to find a non-toxic ORO dyeing solution and optimize the current ORO dyeing method.

Salicylic acid, a fat-soluble organic acid, has been used for disinfection and skin care [13–15]. In this study, we prepared different solvents by mixing different concentrations of salicylic acid and ethanol to dissolve ORO, and compared their ability to dissolve ORO by colorimetry. At the same time, the 0.5% ORO solution in 60% isopropanol was used as a control. The staining effects for different cell lines and adipose tissue sections were compared by semi-quantitative analysis [16,17]. Finally, a salicylic acid ethanol solution (containing 50% ethanol, 5%-10% salicylic acid) for preparation of 0.5% ORO solution was screened out, which has a better staining effect on fat staining in cells and tissues, the stains in fat droplets were bright red to dark red, with a clear background. The staining effect was comparable to, or even superior to, ORO solution in 60% isopropanol. What’s more, this ORO solution is non-toxic and can be stored for a long time. Therefore, the modified ORO staining method can be widely popularized and applied in laboratories.

## Results

### Solubility of ORO in different solvents

**Figure 1.**
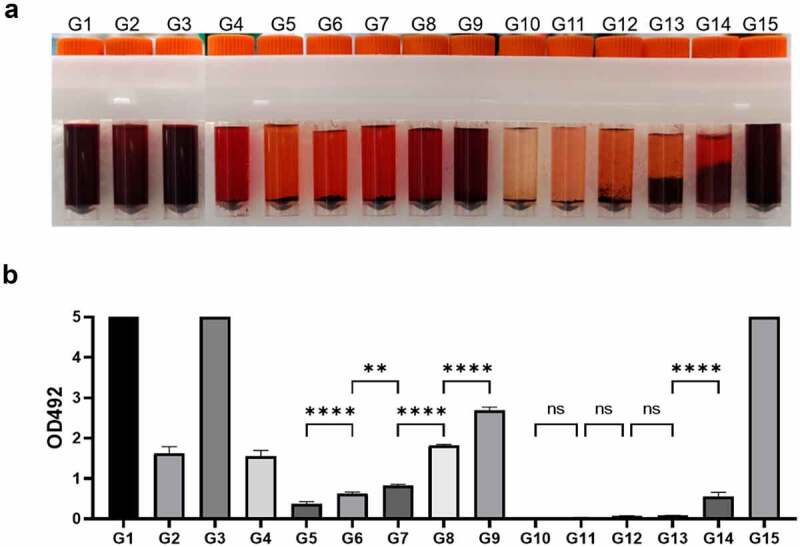
Solubility of ORO in different solvents. **A**: 15 ORO solutions show different degrees of red, representing different ORO dissolving abilities of 15 solvents. **B**. The absorbance at 492 nm of 15 ORO staining solutions semi-quantitatively reflects the solubility of ORO in different solvents. (n = 3. Error bars indicate mean ± SD. ns: p > 0.05, **P < 0.01; ****P < 0.0001, by ANOVA for multiple comparisons).

According to the principle of ORO staining, the concentration of ORO in the dyeing solution will affect the dyeing effect of ORO [4,8]. First of all, we evaluated the dissolution of ORO by the colour of each dye solution. As shown in Figure 1a, the order of colour depth judged by eyes is G1 = G3 = G15> G9 = G2> G8> G4> G7 = G14> G6> G5> G13> G12> G11> G10. At the same ethanol concentration, the solubility of ORO increased with the increasing concentration of salicylic acid. Among these 15 groups, the 30% ethanol + 5% salicylic acid and 30% ethanol + 10% salicylic acid groups had flocculent precipitates, which were insoluble salicylic acid. Adding ethanol to a concentration of more than 50% could dissolve the flocculent precipitates. The solvent containing 70% ethanol + 10% salicylic acid had high solubility of ORO and exhibited redder and brighter colour than that of 100% isopropanol dye solution.

Furthermore, we measured the absorbance of each dye solution at 492 nm to quantitatively compare the ORO content in different dye solutions (Figure 1b). The results were consistent with the trend observed by eyes.

### Staining effect for different cells

**Figure 2.**
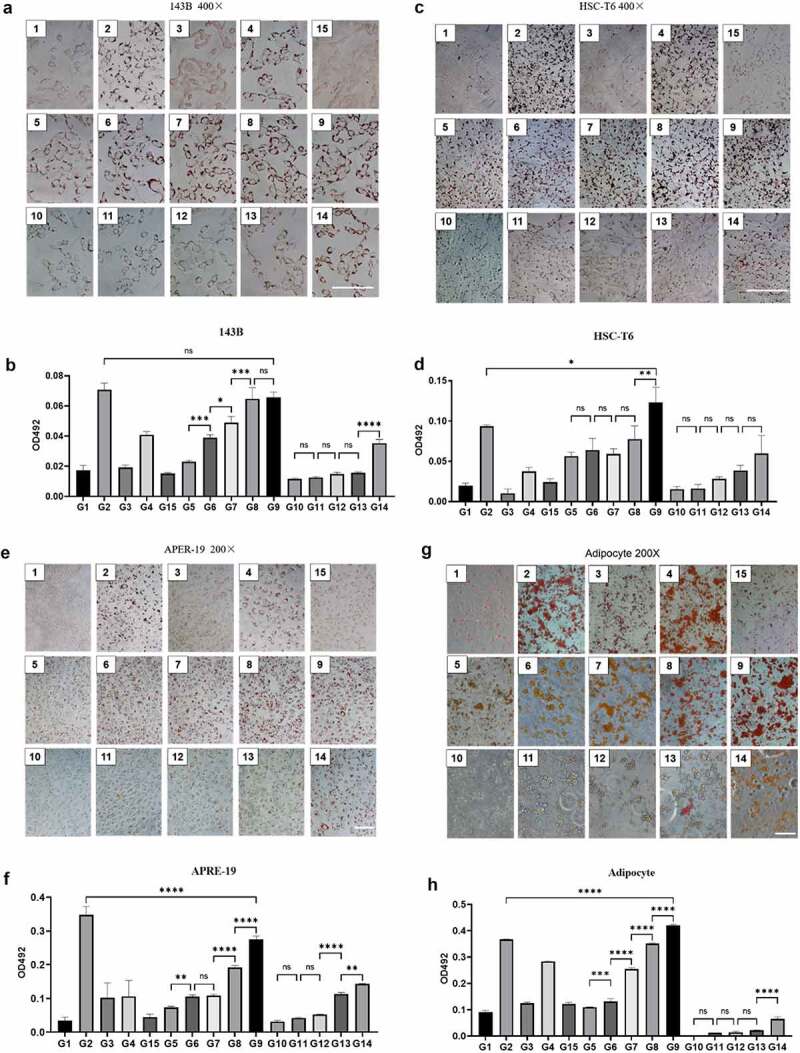
The staining effect of different dye solutions in different cells. **A, C, E, G**: Representative images of ORO staining for 143B human osteosarcoma cells (A), HSC-T6 rat hepatic stellate cells (C), APRE-19 human retinal epithelial cells (E), and adipocyte (G) adipocyte under the light microscope. Scale bars: 100 μm. **B, D, F, H**: The absorbance at 492 nm of ORO-containing extracts semi-quantitatively reflects the staining effect of 15 ORO staining solutions for 4 different cell lines. All the experiment was independently repeated three times. (n = 3. Error bars indicate mean ± SD. ns: p > 0.05, **P < 0.01; ***P < 0.001, ***P < 0.0001, by Student’s t-test between 2 groups comparisons).

Three different cell lines containing fat were chosen for cell staining. Firstly, we compared the staining effect of 15 kinds of ORO staining solutions on 143B human osteosarcoma cells. The staining effect of ORO under the light microscope was shown in Figure 2a, and the dyeing area, as well as the colour of lipid droplets from dark to light, were listed as follows, G2 = G9 = G8> G7> G6 = G14 = G4> G5> G13> G12> G11> G10> G1 = G3 = G15. Except for groups G1, G3, and G15, other ORO solutions could positively stain lipid droplets. The lipid droplets in groups G10, G11, G12, and G13 were lightly stained and yellow, while the lipid droplets in other positive stained groups were orange-red to dark red. At the same ethanol concentration, the staining effects improved with the increase of salicylic acid concentration.

In G8 and G9, the red staining of lipid droplets in the cytoplasm could be observed after 5 minutes of staining, and the best staining effect can be achieved after 10–15 minutes of staining. There was no difference between the red depth of lipid droplets stained for more than 2 hours and overnight (12–16 hours). What is noteworthy is that the background of staining was clean without impurities and red crystals in these two groups.

After photographing and recording, the staining effects of different dye solutions were quantitatively compared according to the method of Jun et al. [18], as shown in Figure 2b. The results were consistent with the trend observed under the microscope.

Next, we repeated the staining process in HSC-T6 rat hepatic stellate cells (Figures 2c and 2d), APRE-19 human retinal epithelial cells (Figures 2e and 2f), and primary mature adipocytes (Figures 2g and 2h). The trend of the staining effect was the same as that of 143B human osteosarcoma cells. There was statistic difference between G9 and G2 in other three cells, the staining effect of G9 was better than that of G2 in HSC-T6 cells and adipocytes, while less than that of G2 in APRE-19 cells, which might due to the experimental batches and cell induction.

Therefore, the ORO staining effect of 50% ethanol + 10% salicylic acid (G9) was the best, which was equivalent to 60% isopropanol (G2), followed by 50% ethanol + 5% salicylic acid (G8). Meanwhile, the staining of small lipid droplets in G9 and G8 was clearer than that in group (2).

### Staining effect for different tissue sections

Lipid-rich tissues, including the liver, aortic arch, skin, and adipose tissue, were collected to detect the staining effect of different ORO solutions.

**Figure 3.**
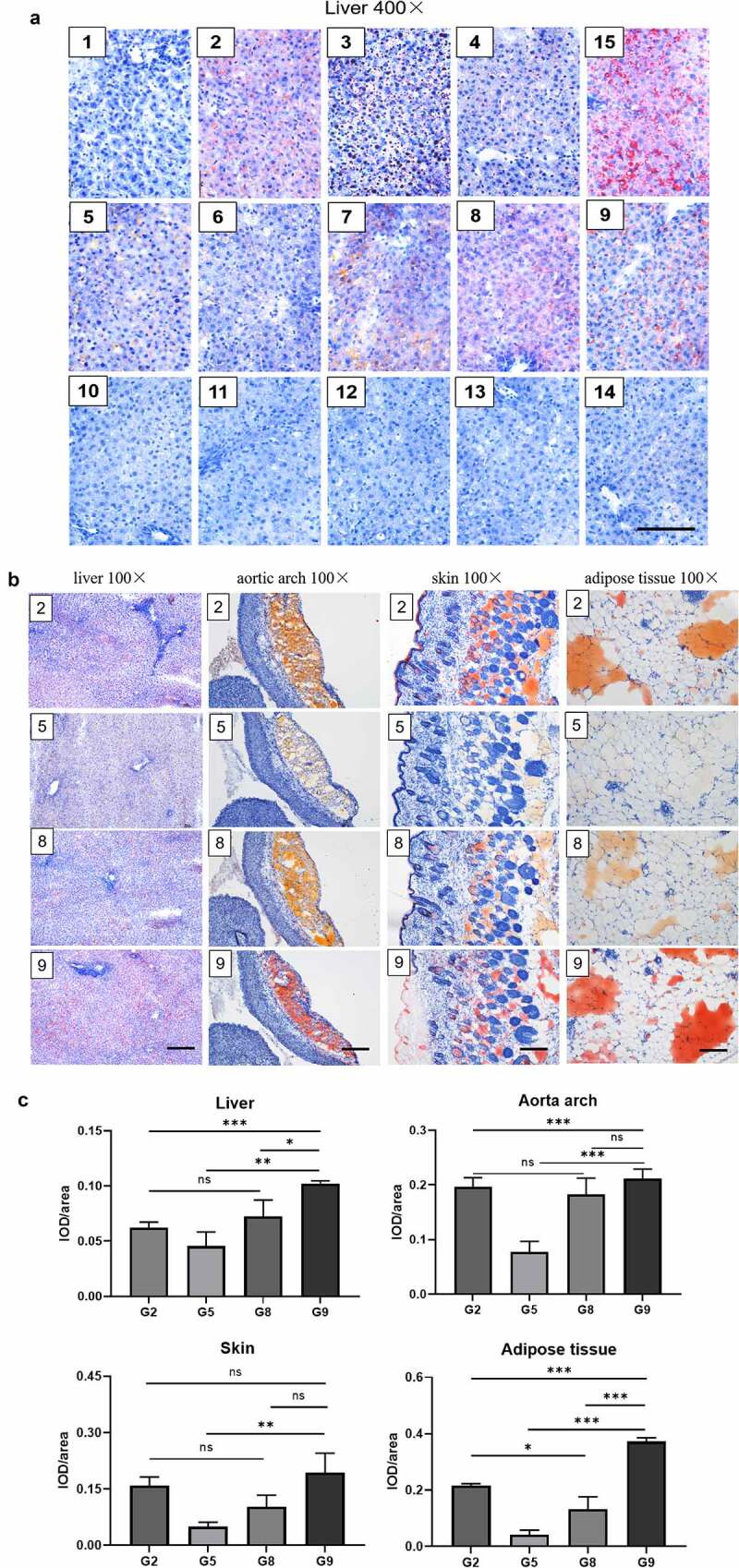
The staining effect of different dye solutions for different tissue sections. **A**: Representative images with 15 kinds of staining solutions for continuous liver sections under the light microscope. Scale bars: 100 μm. **B**: Further staining with staining solutions of groups (2), (5), (8), and (9) for the aortic arch, skin, and adipose tissue sections. Scale bars: 200 μm. **C**: Quantitative comparison of the staining effect of groups (2), (5), (8), and (9) for the liver, aortic arch, skin, and adipose tissue sections. Representative of 3 photographs for every solution group and the mean was estimated. (n = 3. Error bars indicate mean ± SD. ns: p > 0.05, *: p < 0.05; **P < 0.01; ***P < 0.001, by ANOVA for multiple comparisons).

First of all, continuous liver sections were stained with 15 kinds of staining solutions. The staining results were consistent with that of cell staining (Figure 3a). There was almost no staining of lipid droplets in G1, G3, G10, G11, G12, G13, and G14. Lipid droplets in other groups were orange to deep red. The staining background of G8 and G9 was clean, without impurities and red crystals. The staining area and lipid droplet colour from dark to light were G15 > G9 > G2> G8 > G7 > G6 > G4 > G5. In the 100% ethanol (G3), many large black and red bubbles were floating above the tissue section. Red hollow ORO bubbles in the 70% ethanol + 10% salicylic acid (G15) were inconsistent with cell contour, indicating false-positive red staining.

Then, we chose G2, G5, G8, and G9 to repeat staining in the aortic arch, skin, and adipose tissues (Figure 3b). Noteworthy, these four dye solutions are obtained by diluting the storage solutions stored for several weeks and distilled water in a certain ratio. The stained fat in G8 and G2 exhibited orange-red, and the red colour of G2 was deeper than that of G8. While the stained fat in G9 exhibited deep red, a light-yellow stain was observed in G5. A quantitative comparison of the staining effect was performed by Image-Pro Plus software (Figure 3c), and the results were consistent with the trend under the light microscope.

The above results supported that the ORO dye solution prepared with a solvent containing 50% ethanol and 10% salicylic acid had a clean background and a better dyeing effect than the ORO dye solution prepared with 60% isopropanol in tissue staining. At the same time, the storage solution with the easy preparation and harmless process still has a good staining effect.

## Discussion

This study provided a salicylic acid ethanol solution (containing 50% ethanol, 5%-10% salicylic acid) for the preparation of ORO solution, which has a better staining effect on lipid staining in cells and tissues, with a clean background and short dyeing time than commonly used method.

ORO staining is a method to display fat in tissues or cells [2,5,6]. The principle is that the solubility of ORO in neutral lipids is higher than that in the dye solution. During staining, ORO is transferred from the dye solution to fat, so that the lipid droplets can be stained red [3,4]. At present, freshly prepared ORO in 60% isopropanol is the most commonly used method [19,20]. However, it has a deep staining background and poor staining effect for samples with a small number of intracellular lipid droplets and a small volume of lipid droplets [11,12]. Ethanol is a non-toxic organic solvent, which also can be used to dissolve ORO and is harmless to the human body. At present, it has been reported that 60% or 50% ethanol is used to prepare ORO solution [21], but the solubility of ORO in 60% or 50% ethanol is not high, the staining effect is not good, and the lipid staining is light. Under the same conditions, the staining effect is far worse than that of ORO in 60% isopropanol.

To address the shortcomings of current ORO staining methods, we provide a salicylic acid ethanol solvent for the preparation of the ORO solution. Salicylic acid, molecular formula C7H6O3, is a fat-soluble organic acid, soluble in ethanol, acetone, ether, and other organic solvents [22]. As an important raw material, salicylic acid is often used in the field of fine chemicals such as medicine, spices, dyes, and rubber additives [23]. It also has the function of clearing heat, detoxifying, and anti-inflammatory bactericidal [13–15]. Clinically, 2–3% salicylic acid ethanol (containing 70% ethanol) is used to inhibit bacteria and relieve itching. 2%~10% salicylic acid ethanol (containing 70% ethanol) is a commercial reagent in cosmetics industry, and also dissolve the oil in pores, shrink pores and blackhead acne, exfoliate aged horniness of skin without toxic side effects on the human body [24–27].

Specifically, the solvent containing 30% or 50% ethanol and 1%-10% salicylic acid in different proportions was prepared, and then 0.5% ORO dye solution was dissolved with these solvents. At the same ethanol concentration, the solubility of ORO increased with the increase of salicylic acid concentration. Because salicylic acid is insoluble in water, when the concentration of ethanol decreases, a flocculent precipitate will form in high concentration salicylic acid-containing groups. Adding ethanol to 50% concentration can completely dissolve 10% salicylic acid. For the selection of cell lines, we used three types of cells commonly used in the laboratory as well as primary adipocytes, which were stained after different treatments. To be specific, 143B is a human osteosarcoma cells line, liposarcoma of bone is a malignant tumour with a fatty phenotype, and positive ORO stains can be observed in 143B or other osteosarcoma cells [28,29]. The APRE-19 human retinal epithelial cells [30,31] were induced by sodium oleate for 48 hours to be positively stained with ORO. The physiological functions of hepatic stellate cells in the liver are mainly involved in the function of vitamin A metabolism and storing fat, the quiescent hepatic stellate cells are rich in fat droplets [32,33]. HST-T6 hepatic stellate cell line was induced with 100 μmol/L sodium oleate and 10 μmol/L all-trans retinoic acid for 3–5 days to be returned to its quiescent state.

In 100% isopropyl alcohol, 100% ethanol, and 70% ethanol+10% salicylic acid solvents, the solubility of ORO were very high, but the cell staining effect was not good, and almost no red stains could be observed in lipid droplets. There was also no positive staining but floating ORO blobs in tissue sections of the above three groups. These results indicated that the high solubility of ORO in these solvents was comparable to, or even exceeded the solubility of ORO in fat, which would result in a bad staining effect and high background.

We tried to appropriately increase the solubility of ORO by decreasing ethanol concentration and increasing salicylic acid concentration, which also could reduce the disadvantage of organic solvent volatilization resulting in short solution storage time. In the 30% ethanol group, the basic solubility of ORO was low, 10% salicylic acid could not be dissolved and the flocculent precipitate was precipitated. So that the staining effect of 30% ethanol groups on cells and tissues was not good, and the lipid droplets were light colour. In the 50% ethanol group, the solubility of ORO increased with the increase of salicylic acid concentration. The staining effect of 50% ethanol +5% salicylic acid group and 50% ethanol +10% salicylic acid group on fat in cells was similar. But in the tissue staining, the lipid stains of 50% ethanol +10% salicylic acid group showed bright red, significantly better than the orange-red colour of 50% ethanol +5% salicylic acid group, while the fat staining of 50% ethanol group without salicylic acid was light yellow stained. This result may be due to the much higher content of lipid droplets in tissue sections compared to cells, which can absorb more ORO from the staining solution. Therefore, as the ORO content in the staining solution increases, the staining effect will be correspondingly better.

To further reduce the use of harmful isopropanol, we replaced the 60% isopropanol with 50% ethanol in the dehydration step before cell staining and the elution step. If cells were eluted with 70% ethanol, 70% ethanol could partially dissolve ORO in the lipid droplets, and the staining colour became lighter than that before elution. In tissue staining, 50% ethanol was not easy to elute impurities of ORO, so 70% ethanol was used for elution, and the staining effect was not significantly different from that before elution and also with a cleaner background.

Although the solution with salicylic acid and ethanol for the preparation of ORO dyes has a number of advantages, several aspects need to be improved. Firstly, it is inevitable that the ethanol used for ORO dye preparation also has the problem of volatilization, but compared with isopropanol, this method greatly reduces the use of toxic chemical reagents during the operation. Secondly, since salicylic acid is insoluble in water, some salicylic acid crystals will be precipitated with the volatilization of ethanol during the dyeing process. But these crystallized salicylic acids can be washed out during the differentiation step and will not have much effect on the staining results.

For the convenience of follow-up experiments, we also prepared saturated ORO solution with 100% ethanol and 20% salicylic acid. After 6 months, the ORO solution was 1:1 diluted by ddH_2_O to get the ORO working solution, its staining effect was not different compared to freshly prepared ORO solution in 50% ethanol and 10% salicylic acid. The saturated ORO solution with 100% ethanol and 20% salicylic acid could be stored for a long time to be used as a commercial reagent.

## Conclusions

In summary, this study provides a modified ORO staining solution containing 0.5% ORO, 50% ethanol, and 5–10% salicylic acid, which can effectively stain fat in different cells and tissues with a cleaner background and short dyeing time. The staining effect is comparable to, or even better than that of 0.5% ORO solution prepared with 60% isopropanol. At the same time, the solution is non-toxic, convenient to prepare, and can be stored for a long time. Therefore, the modified ORO staining solution and method can be widely promoted and applied in laboratories.

## Materials & methods

### Preparation of different solvents and ORO dye solutions

To compare the staining effects of the ORO staining solution prepared with different concentrations of salicylic acid ethanol solution, we prepared 15 different solvents. Firstly, 20% salicylic acid ethanol solution (100% ethanol + 20% salicylic acid) was prepared by dissolving 200 g salicylic acid powder in 1000 ml of 100% ethanol. Different solutions(G1-G15) were prepared with distilled water, isopropanol (Chengdu Chron chemical reagent Co., Ltd. Chengdu, Sichuan, China), ethanol (Chengdu Chron chemical reagent Co., Ltd), and salicylic acid powder (Tianjin Fuchen chemical reagent Co., Ltd. Tianjin, China) as described in Table 1. Among them, 60% ORO isopropanol was set up as positive control. 100% isopropanol and 100% ethanol were set up as solubility control due to their high solubility for ORO dye. Then, 0.2 g of ORO powder (Sigma-Aldrich, St. Louis, MO, USA) was dissolved in 40 ml of the above solvents to prepare 0.5% ORO solutions which were followed by being blown with a pipet evenly and placed for 10 minutes. Next, the solubility was preliminarily evaluated by the colour of the solutions. After that, the ORO solutions were filtered by filter paper or 0.45 μm filter, and 50 μl of each solution was put into a 96-well plate to detect the OD value at 492 nm wavelength with a microplate reader [18].

**Table 1.** Composition of different solvents for ORO preparation.

Group	Solvents	Formula (per 100 ml)			
		100% isopropanol	100% ethanol	100% ethanol +20% salicylic acid	ddH2O
G1	100% isopropanol	100 ml	0 ml	0 ml	0 ml
G2	60% isopropanol	60 ml	0 ml	0 ml	40 ml
G3	100% ethanol	0 ml	100 ml	0 ml	0 ml
G4	70% ethanol	0 ml	70 ml	0 ml	30 ml
G5	50% ethanol	0 ml	50 ml	0 ml	50 ml
G6	50% ethanol+1% salicylic acid	0 ml	45 ml	5 ml	50 ml
G 7	50% ethanol+2% salicylic acid	0 ml	40 ml	10 ml	50 ml
G 8	50% ethanol+5% salicylic acid	0 ml	25 ml	25 ml	50 ml
G 9	50% ethanol+10% salicylic acid	0 ml	0 ml	50 ml	50 ml
G 10	30% ethanol	0 ml	25 ml	5 ml	70 ml
G 11	30% ethanol+1% salicylic acid	0 ml	30 ml	0 ml	70 ml
G 12	30% ethanol+2% salicylic acid	0 ml	20 ml	10 ml	70 ml
G 13	30% ethanol+5% salicylic acid	0 ml	5 ml	25 ml	70 ml
G 14	30% ethanol+10% salicylic acid	0 ml	30 ml	+ 10 g salicylic acid powder	70 ml
G 15	70% ethanol+10% salicylic acid	0 ml	20 ml	50 ml	30 ml

After screening out the suitable concentration, we routinely configured three storage solutions for long-term storage in order to facilitate use and storage. The storage solution is a saturated solution obtained by dissolving ORO with 100% ethanol, 100% ethanol containing 10% salicylic acid, and 100% ethanol containing 20% salicylic acid, which can be used by simply diluting it 1:1 with distilled water to obtain the working solution of G5, G8 and G9. Subsequent staining of different tissues was performed using these storage solutions.

### Cell culture and treatment

Three adipose-containing cell lines were used to compare the staining effects of different ORO solutions: human osteosarcoma cell 143B, rat hepatic stellate cell HSC-T6, and human retinal epithelial cell APRE-19, all of which were purchased from the American Type Culture Collection (Manassas, USA). Cells were plated into a 48-well plate and cultured in Dulbecco’s modified Eagle’s medium (DMEM, Gibco, Carlsbad, USA) supplemented with 10% foetal bovine serum (FBS, Gibco, Carlsbad, USA), 100 U/mL penicillin, and 100 µg/mL streptomycin (Gibco, Carlsbad, USA) with 5% CO2 at 37°C with 5% CO2. 143B cells were seeded with an initial cell density of 40% and cultured for 24 h after adhesion. HSC-T6 cells were seeded with an initial cell density of 15–20%. After cell adhesion, the medium was changed into DMEM complete medium containing 100 μmol/L sodium oleate and 10 μmol/L all-trans retinoic acid for 3–5 days. APRE-19 cells were seeded with an initial cell density of 20–30%. After cell adhesion, the medium was changed into DMEM/F12 complete medium containing 100 μmol/L sodium oleate for 48 hours. Primary adipocytes were extracted from fat tissue of 5-week old rat and cultured for three days by using a modified ceiling culture method [34], the slide with adherent adipocytes was put in 12-well plated and fixed for staining. The above cells will be used randomly for subsequent staining.

### ORO staining of cell

At the indicated time point, the medium was removed, and cells were washed with PBS and fixed in 4% paraformaldehyde for 20–30 minutes, followed by washing with PBS three times. After PBS was removed, 500 μl of 50% ethanol was added and washed for 15–20 seconds to remove water. Following that, 200 μl of freshly prepared ORO solutions according to Table 1 were added to the wells respectively and incubated for 10–15 minutes at room temperature. ORO solution was pipetted out, cells were washed with 500 μl of 50% ethanol for 15–20 seconds to remove excess dye solution, and then washed with PBS for more than 3 times until the liquid is clarified, pictures were captured under a microscope within 24 h before quantitative analysis. In the dehydration step and elution step, 50% ethanol was replaced by 60% isopropanol in group 1 and group 2.

Furthermore, the staining effects of different solutions in cells were quantitatively analysed according to the reported method [18]. Briefly, after complete washing and drying, 100 μl of 100% ethanol was added to each well and incubated on a shaker for 10 min at room temperature to release the ORO from the stained cells. Finally, 50 μL of the ORO-containing extract was transferred to a 96-well plate, and the absorbance at a wavelength of 492 nm was measured with a microplate reader. All the experiment was independently repeated at least three times.

### Animals and ethical statement

All procedures involving animals were reviewed and approved by the Animal Care and Use Committee of Chongqing Medical University. Two SPF Sprague-Dawley rats (5 weeks old, female, qualified number: SCXK (Beijing) 2014–0004) were purchased from the Tengxin Institute of Biotechnology (Chongqing, China) and kept at the Laboratory Animal Center, Children’s Hospital of Chongqing Medical University (Animal Experiment Licence: SYXK 2017–0012). They were given free access to water, food, and housed in a well-ventilated, relative humidity 50%-70%, temperature-controlled room (22°C) with 12 hours’ light and dark cycles.

### Preparation of tissue samples

All rats were fed with a 60% high-fat diet for at least 2 weeks and then euthanized humanely using CO_2_ by inhalation (100% CO_2_) followed by cervical dislocation. Next, the liver, aorta, skin, and adipose tissue were collected and rapidly frozen in liquid nitrogen-cooled isopentane and embedded in Tissue-Tek. Sections of each tissue sample were cut to a thickness of 12 μm, then air-dried and fixed with 4% formalin for 30 min before processing to avoid detachment.

### ORO staining of tissue

For the tissue sections, staining was randomly performed according to the method of Du et al. [35]. Briefly, the sections prepared above were rinsed with tap water for 1 min and dehydrated with either 60% isopropanol or 70% ethanol and then stained with different freshly prepared ORO solutions for 30 min in the dark respectively. After ORO staining, sections were quickly rinsed with 60% isopropanol or 70% ethanol, followed by multiple rinses with ddH_2_O until the solution was clear, then counterstained with 10% Mayer’s haematoxylin. Following that, the sections were rinsed three times with tap water and once with ddH_2_O, dried, fixed with Kaiser’s glycerine gelatin (Merck, Germany), and photographed under an inverted microscope.

Semi-quantitative analysis was performed according to the method of Wang et al. [17]. Under the same shooting conditions, 3 fields of view under high magnification were selected randomly and processed by the Image processing program Image-Pro Plus 6.0 (Media Cybernetics, CA, USA) which counts the values of IOD sum and area sum in the same area in each photograph. Then, the values of IOD/Area (Average Optical Density, AOD) were used for statistical analysis, and the mean was estimated.

### Statistical analysis

All data were presented as mean± standard deviation (SD) and analysed using the Prism 8 software (GraphPad) package. The statistical significance of the differences between groups was assessed using a two-tailed Student’s t-test for pair-wise comparisons, or a one-way analysis of variance followed by a post hoc Student-Newman-Keuls test for multiple comparisons. A p < 0.05 was considered to be statistically significant.

## Supplementary Material

Supplemental Material

## Data Availability

The authors confirm that the raw data supporting the findings of this study are available within the article (and/or) its supplementary materials which can be accessed online at https://doi.org/10.1080/21623945.2023.2179334
