# Supplementary material for: An optimized method for Oil Red O staining with the salicylic acid ethanol solution
Source: Adipocyte. 2023 Feb 24;12(1):2179334. doi: 10.1080/21623945.2023.2179334 (PMC9980477; doi:10.1080/21623945.2023.2179334)
Supplement: Supplemental Material [file KADI_A_2179334_SM8752.zip › Supplementary File_Table 1.docx]

**Table 1.** **Composition of different solvents for ORO preparation**

| Group | Solvents | Formula (per 100ml) | | | |
| --- | --- | --- | --- | --- | --- |
|  |  | 100% isopropanol | 100% ethanol | 100% ethanol  +20% salicylic acid | ddH2O |
| G1 | 100% isopropanol | 100 ml | 0 ml | 0 ml | 0 ml |
| G2 | 60% isopropanol | 60 ml | 0 ml | 0 ml | 40 ml |
|  |  |  |  |  |  |
| G3 | 100% ethanol | 0 ml | 100 ml | 0 ml | 0 ml |
| G4 | 70% ethanol | 0 ml | 70 ml | 0 ml | 30 ml |
|  |  |  |  |  |  |
| G5 | 50% ethanol | 0 ml | 50 ml | 0 ml | 50 ml |
| G6 | 50% ethanol+1% salicylic acid | 0 ml | 45 ml | 5 ml | 50 ml |
|  |  |  |  |  |  |
| G 7 | 50% ethanol+2% salicylic acid | 0 ml | 40 ml | 10 ml | 50 ml |
| G 8 | 50% ethanol+5% salicylic acid | 0 ml | 25 ml | 25 ml | 50 ml |
| G 9 | 50% ethanol+10% salicylic acid | 0 ml | 0 ml | 50 ml | 50 ml |
| G 10 | 30% ethanol | 0 ml | 25 ml | 5 ml | 70 ml |
| G 11 | 30% ethanol+1% salicylic acid | 0 ml | 30 ml | 0 ml | 70 ml |
| G 12 | 30% ethanol+2% salicylic acid | 0 ml | 20 ml | 10 ml | 70 ml |
| G 13 | 30% ethanol+5% salicylic acid | 0 ml | 5 ml | 25 ml | 70 ml |
| G 14 | 30% ethanol+10% salicylic acid | 0 ml | 30 ml | + 10g salicylic acid powder | 70 ml |
| G 15 | 70% ethanol+10% salicylic acid | 0 ml | 20 ml | 50 ml | 30 ml |

Table 1. Composition of different solvents for ORO preparation
