# Supplementary figures and images for: An optimized method for Oil Red O staining with the salicylic acid ethanol solution
Source: Adipocyte. 2023 Feb 24;12(1):2179334. doi: 10.1080/21623945.2023.2179334 (PMC9980477; doi:10.1080/21623945.2023.2179334)

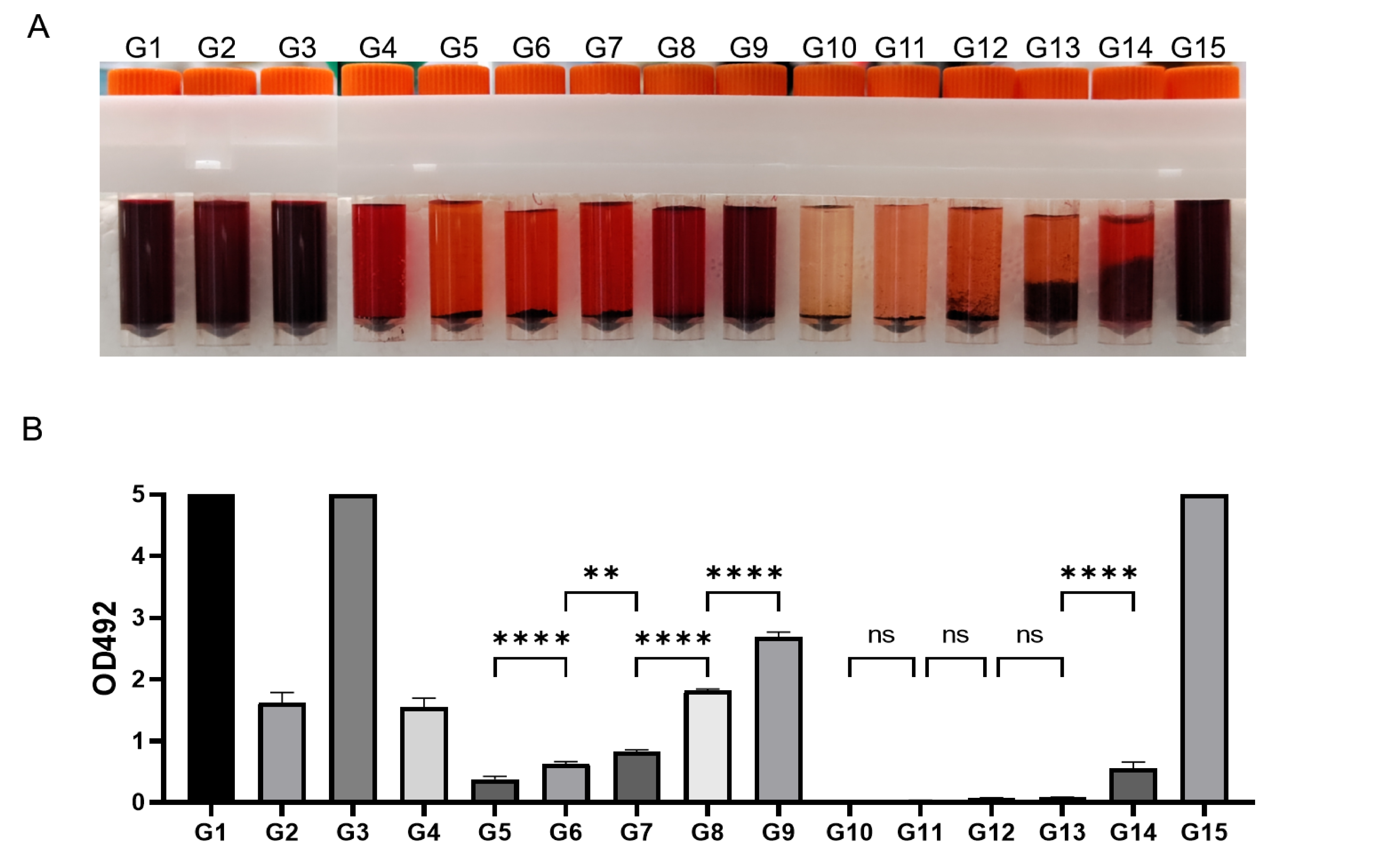

Supplement: Supplemental Material [file KADI_A_2179334_SM8752.zip › Supplementary File_Figure 1.tif]

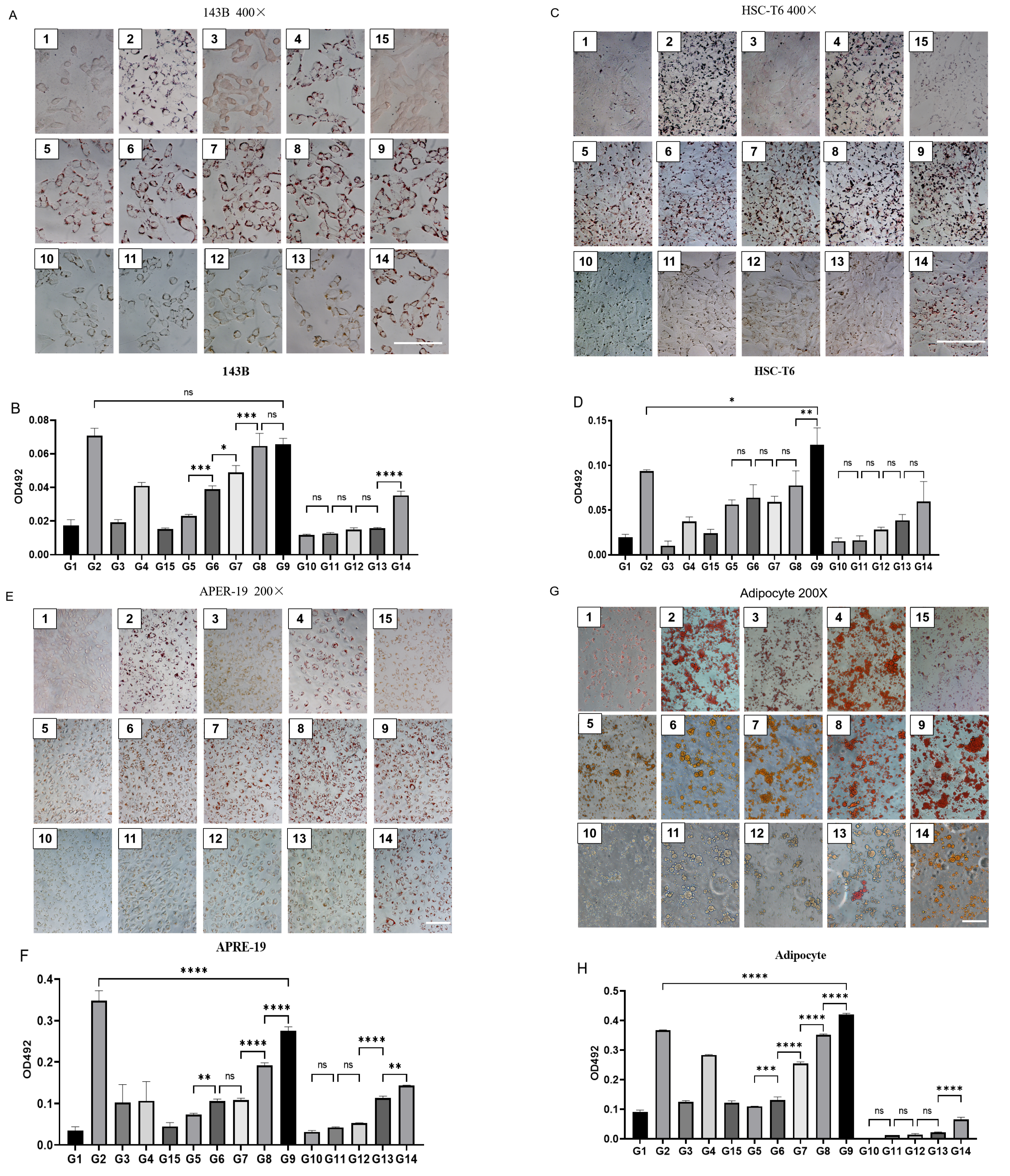

Supplement: Supplemental Material [file KADI_A_2179334_SM8752.zip › Supplementary File_Figure 2.tif]
